# Venous Thromboembolism: Classification, Risk Factors, Diagnosis, and Management

**DOI:** 10.5402/2011/124610

**Published:** 2011-10-17

**Authors:** Fatemeh Moheimani, Denise E. Jackson

**Affiliations:** Thrombosis and Vascular Diseases Laboratory, Health Innovations Research Institute and School of Medical Sciences, RMIT University, P.O. Box 71, Bundoora, VIC 3083, Australia

## Abstract

Venous thromboembolism (VTE) is categorised as deep venous thrombosis (DVT) and pulmonary embolism (PE). VTE is associated with high morbidity and causes a huge financial burden on patients, hospitals, and governments. Both acquired and hereditary risks factors contribute to VTE. To diagnose VTE, noninvasive cost-effective diagnostic algorithms including clinical probability assessment and D-dimer measurement may be employed followup by compression ultrasonography for suspected DVT patients and multidetector computed tomography angiography for suspected PE patients. There are pharmacological and mechanical interventions to manage and prevent VTE. The pharmacological approaches mainly target pathways in coagulation cascade nonspecifically: conventional anticoagulants or specifically: new generation of anticoagulants. Excess bleeding is one of the major risk factors for pharmacological interventions. Hence, nonpharmacological or mechanical approaches such as inferior vena cava filters, graduated compression stockings, and intermittent pneumatic compression devices in combination with pharmacological interventions or alone may be a good approach to manage VTE.

## 1. Introduction

Venous thromboembolism (VTE) is a major health and financial burden that affects the community [1]. About 30000 Australian hospitalisations may be caused by VTE that result in losing life of 5000 patients each year [2]. This condition is the third most common vascular disorder in Caucasian populations after myocardial infarction and stroke [3]. VTE is an acute event which was estimated to complicate 2-3 per 1000 hospital admissions followed by principle diagnosis [1]. VTE presents clinically as deep venous thrombosis (DVT) and pulmonary embolism (PE) with serious outcomes in both men and women [3]. However, most of these complications and deaths are preventable with appropriate administration of cost-effective antithrombotic drugs and nonpharmacological interventions [2]. There are various strategies to investigate suspected VTE including clinical pretest probability combined with/without measurement of D-dimer, known as algorithm strategies, and imaging techniques [4]. In this paper we have summarised several aspects of VTE including different classifications, potential risk factors, various diagnostic methods, and prevention and treatment interventions.

## 2. Classifications

### 2.1. Deep Venous Thrombosis (DVT)

DVT usually initiates in the calf area of the leg. The majority of thrombi form in the deep veins below the popliteal trifurcation (distal DVT) most likely to resolve spontaneously with no symptoms [5, 6]. About 60–70% of patient with symptomatic VTE develop DVT [7]. Most patients present with symptoms when distal DVT extend to the popliteal and femoral veins and other proximal vein [5, 6]. DVT can lead to complications such as postphlebitic syndrome, PE, and death [4]. There is a 50% chance that patients with untreated symptomatic proximal DVT develop symptomatic PE within 3 months [5, 6]. An important complication of DVT is postthrombotic syndrome that develops in 20–50% of patients and may result in lifelong limb pain, swelling, heaviness, oedema, and leg ulcers [8, 9]. DVT reoccurs in about 10% of patients who may develop severe postthrombotic syndrome within 5 years [5, 6].

### 2.2. Pulmonary Embolism (PE)

PE symptoms, such as new or worsening dyspnoea, chest pain, or sustained hypotension with no alternative cause [10], occur in about 30–40% of patients with VTE [7, 11]. The survival rate for patients with PE is worse than DVE as the sudden death is the initial clinical presentation of 25% of these patients [12]. When this condition is diagnosed in patients, with no further treatment the fatality rate can reach 25% [13]. However, prescription of anticoagulant reduces this risk to 1.5% [14].

## 3. Risk Factors

Both acquired and hereditary factors play essential roles in development of VTE [15–17]. The acquired risk factors for VTE are categorised as strong (odds ratio >10), moderate (odds ratio 2–9), and weak (odds ratio <2) [15]. Fracture (hip or leg), hip or knee replacement, major general surgery, major trauma, and spinal cord injury are considered as strong risk factors [15]. Moderate risk factors include arthroscopic knee surgery, central venous lines, chemotherapy, congestive heart or respiratory failure, hormone replacement therapy, malignancy, oral contraceptive therapy, paralytic stroke, pregnancy/postpartum, previous VTE, and thrombophilia [15]. Whereas bed rest (>3 days), extended immobility (air travel >8 hours), increasing age (≥40 years), laparoscopic surgery, obesity, pregnancy/antepartum, and varicose veins are considered as weak risk factors [6, 15]. 

A variety of inherited factors contribute to VTE as well [15–17]. These are also known as strong, medium and weak genetic risk factors [17]. Deficiencies of some natural coagulation inhibitors including antithrombin (AT), protein C (PC), and its cofactor protein S (PS), insufficiency of anticoagulant pathways such as tissue factor pathway inhibitor (TFPI), thrombomodulin and endothelial protein C receptor (EPCR) [16, 17], and elevated level of factor VIII [18, 19] belong to strong genetic risk factors. Moderate genetic risk factors consist of mutation in the factor V Leiden (FVL) causing resistance to activated protein C (APC-resistance), a mutation in the 3′-untranslated part of the prothrombin (Factor II) gene (prothrombin 20210A, rs 1799963) which results in increased prothrombin levels, blood group (non-O blood group), and a C- to T-variation at position of 10034 in the fibrinogen gamma chain (rs 2066865) leading to reduction of the fraction of gammafibrinogen in plasma [17]. Risk factors, with relative risk 1.0–1.5, such as mutation in C > T at position 677 (rs1801133) methylenetetrahydrofolate reductase (MTHFR) resulting in minor elevation of homocysteine levels [17, 20], and homozygous factor XIII 34Val alleles are categorised as weak genetic risk factors [21].

## 4. Diagnosis of VTE

The initial diagnostic step for determination of VTE is the clinical probability assessment [24]. For suspected DVT, the Wells score has well-established criteria (Table 1) [22]. Based on this clinical model, pre-test probability may predict for patients with clinical characteristics described in Table 1 [22, 23]. A score ≥2 indicates that the probability of DVT is likely, and a score of <2 indicates that the probability of DVT is unlikely [23]. The Wells score for suspected PE is listed in Table 2 [24]. A score >4 indicates PE likely, and a score of ≤4 indicates PE unlikely [24]. The limitation of Wells' scoring system may be subjective nature of each criterion and its reliability on the physicians' judgment [25]. Other scoring systems to predict PE are revised Geneva and simplified revised Geneva (Table 2) [24–26]. Revised Geneva score is based on clinical variable and independent from physicians' judgement (Table 2) [25]. To prevent miscalculations in an acute setting, simplified revised Geneva score was designed with no defeat in diagnostic accuracy and clinical utility (Table 2) [26]. However, the Wells score reported to be more accurate than simplified revised version of the Geneva score for PE assessment [27].

Clinical probability can subsequently be combined with determination the level of D-dimer, a degradation product of a crosslinked fibrin blood clot [23]. Although these systems perform well in predicting pre-test DVT probability in patients with proximal DVT and outpatients, they are not as sensitive in hospitalised and patients with isolated distal DVT [28]. These initial steps allow selection of patients who requires noninvasive imaging techniques [28] such as compression ultrasonography and venous ultrasound which has replaced venography to diagnose DVT [24, 29]. However, for pelvic vein, venous ultrasound is not as accurate as lower limb DVT due to a limited acoustic window [29]. Hence, computed tomographic (CT) venography may be a good alternative for this condition [29]. Pulmonary CT angiography has also replaced ventilation perfusion scintigraphy of the lung or conventional pulmonary angiography [24, 29]. It has also been reported that combination of this technique with CT venography provides higher sensitivity in detection PE [30]. In addition, it has been shown that transcranial Doppler ultrasonography technique which is based on detection DVT and high-intensity transient signals may screen patients for PE after orthopaedic surgery [31].

## 5. Prevention and Treatment

To prevent thrombus extension, decrease of the risk of recurrent thrombosis and subsequent death in patient with VTE pharmacological and/or mechanical approaches can be administered [8, 32, 33]. 

### 5.1. Pharmacological Interventions

**Figure 1:**
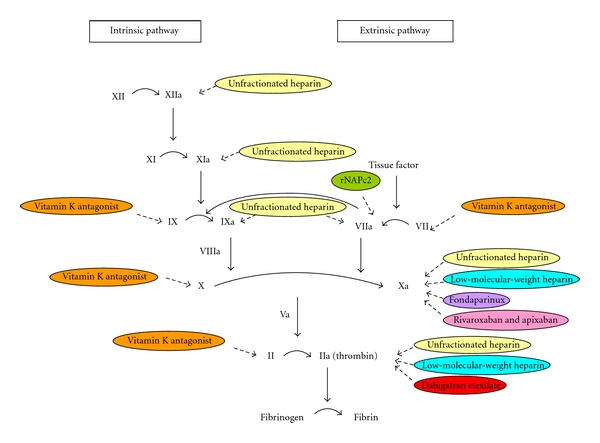
Different pharmaceutical interventions for VTE target various steps in coagulation cascade. Traditional anticoagulants including unfractionated heparin, low-molecular-weight heparin, and vitamin K antagonists target different steps. Despite, new generation of anticoagulants; rNAPc2 (recombinant nematode anticoagulant protein c2), fondaparinux, rivaroxaban, apixaban, and dabigatran etexilate, have specific targets in coagulation pathways.

The pharmacological approaches mainly include a wide range of traditional and new generations of anticoagulants listed in Figure 1.

#### 5.1.1. Conventional Anticoagulants for Treatment of VTE

**(1) Unfractionated Heparin (UFH)**

The initial and standard pharmacological approach in patient with VTE was unfractionated heparin (intravenous: i.v.) (UFH) followed by long-term warfarin [32]. UFH is a heterogenous mixture of glycosaminoglycans that plays its anticoagulation roles via binding to antithrombin by a pentasaccharide, catalysing the inactivation of thrombin and other clotting factors [34]. In addition, UFH has high nonspecific binding affinities to endothelial cells, platelet factor 4, and platelets that result in unpredictable pharmacokinetics and pharmacodynamic properties [34]. Therefore, UFH required laboratory monitoring and has major side effects such as bleeding complications, immune thrombocytopenia, and osteoporosis [32, 34].

**(2) Low-Molecular-Weight Heparin (LMWH)**

UFH has been replaced with subcutaneous administered low-molecular-weight heparin (LMWH), for example, enoxaparin, a derivative of heparin which is polysulfated glycosaminoglycan and has about one-third the molecular weight of UFH [32, 34, 35]. LMWH is as effective as UFH but safer and can be administered in a fixed, weight-adjusted dose [32, 34].

**(3) Vitamin K Antagonists**

Vitamin K antagonists, such as warfarin, are the most common oral anticoagulants for prevention and treatment of VTE; However, they have several disadvantages such as a slow onset of action, a narrow therapeutic window, food and drug interactions, and wide interindividual dosing differences; hence, they are requited intensive monitoring [36]. Therefore, management of patients on these chronic oral anticoagulants encounter difficulties, for example, warfarin resistance and the optimal warfarin initiation dose [36, 37].

#### 5.1.2. New Generation of Anticoagulants for Treatment of VTE

To increase the safety and efficacy of anticoagulants that are more convenient for patients, new generation of oral anticoagulants have been developed [32, 35]. These anticoagulants have been developed from hematophagous organisms via the application of recombinant DNA technology or by structure-based drug design [35]. These anticoagulants target specific steps in the coagulation cascade such as factor VIIa/tissue factor, factor Xa, activated protein C and soluble thrombomodulin, and thrombin [32, 35]. They have been shown to be effective long-term treatment of VTE in phase II and III trials which may be a potential alternative for warfarin [32]. Some of anticoagulants of the new generation with good potential in treatment of VTE are listed below (Figure 1).

**(1) Nematode Anticoagulant Protein c2 (NAPc2)**

Nematode anticoagulant protein c2 (NAPc2) is an inhibitor of factor VIIa/tissue factor complex pathway. NAPc2 is a natural protein initially isolated from canine hookworm and has currently been produced in a recombinant from (rNAPc2) [32, 35]. rNAPc2 forms a complex by binding to a noncatalytic site on both factors X and/or Xa which directly inhibits factor VIIa/tissue factor complex [32, 35]. It has been demonstrated the rNAPc2 is as safe and effective as LMWH for prevention of VTE in patients after elective, unilateral total knee replacement [32, 36, 38].

**(2) Fondaparinux**

Fondaparinux indirectly inhibits factor Xa via binding to AT [32]. Fondaparinux is a synthetic analogue of the pentasaccharide sequence that binds to AT, in UFH and LMWH structure, noncovalently and reversibly. This induces a conformational alteration that enhances the affinity of AT for factor Xa resulting in 300-fold increase in its inhibitory effect [32]. The efficacy of fondaparinux depends on the circulating level of AT, and it cannot be administered orally [39]. It has been demonstrated that Fondaparinux administrated once daily subcutaneously (s.c.) is as effective and safe as adjusted dose of UFH (i.v.) and body weight-adjusted LMWH (s.c.) in the initial treatment of PE and DVT, respectively, [40, 41]. Therefore, Fondaparinux can be a good alternative for LMWH.

**(3) Rivaroxaban**

Rivaroxaban is an oral direct inhibitor of factor Xa [39]. Rivaroxaban inhibits factor Xa in a concentration-dependent manner via a rapid and reversible binding [39]. It has been reported that rivaroxaban reduces the rate of development of VTE in patients after total hip or knee arthroplasty compared with LMWH with no significant differences in risk of bleeding [42–45]. Rivaroxaban has also shown to reduce costs associated with drug administration for prophylaxis and treatment of VTE events in this population as compared with enoxaparin. Rivaroxaban reduces the incidence of symptomatic VTE as well [46]. Therefore, rivaroxaban may be an answer to unmet need for replacement of warfarin as well as a good alternative for LMWH.

**(4) Apixaban**

Apixaban is a reversible active direct inhibitor of factor Xa that can also be administered orally [47]. Prescribing apixaban after knee and hip replacement was more effective for prevention of VTE as compared with LMWH without enhancing bleeding risk [48, 49]. In addition fixed-dose orally administered apixaban may replace LMWH combined with vitamin K antagonists in treatment of DVT [47].

**(5) Dabigatran Etexilate**

Dabigatran etexilate is a competitive reversible oral anticoagulant that inhibits thrombin directly after conversion to its active form dabigatran. Dabigatran etexilate has the potential to replace traditional anticoagulants for prevention of VTE in patients undergone elective total hip or knee replacement surgery [50, 51].

### 5.2. Mechanical Approaches

Nonpharmacological interventions, including graduated compression stockings, intermittent pneumatic compression devices, and inferior vena cava filters, have the advantage of management of VTE with no risk of bleeding [52, 53]. 

#### 5.2.1. Graduated Compression Stockings

Graduated compression stockings apply greater pressure at the ankle than higher up the leg, therefore, reduce pooling of blood in the deep veins [52] which can enhance the velocity of blood outflow toward the heart [54]. It is recommended that patients with acute proximal DVT treated with LMWH should walk with compression bandage or medical compression stockings to assist the recovery from pain and swelling [55]. It has been reported that below-knee compression elastic stockings reduces the risk of postthrombotic syndromes by approximately 50% in patients with proximal DVT [56]. Therefore, it has been recommended that graduated elastic compression stockings with pressure of 30–40 mm Hg at the ankle for 2 years after DVT diagnosis may prevent postthrombotic syndrome [57]. However, there are controversial reports in regards to the efficiency of graduated compression stocking on prevention of VTE in patients. Thigh-level stockings are shown not to be very effective in preventing VTE, and below-knee stockings might even enhance thrombosis in patients with acute stroke [58, 59]. This discrepancy may occur due to initiation of VTE in patients with different diseases. While these stockings may not be as effective in patients with stroke, they may be a good approach in other patients. Hence, future investigation is required to determine the role of these mechanical methods in prevention of VTE.

#### 5.2.2. Intermitted Pneumatic Compression Devices

Intermitted pneumatic compression devices function by cyclic inflation and deflation that promote venous return [52]. While there are no venographic data available confirming the efficacy of graduated compression stockings, intermitted pneumatic compression devices have shown excellent efficacy in several venographic studies over the past 25 years [54]. Intermitted pneumatic compression devices, including single chamber, multiple chamber, calf-length, thigh-length, foot only, and foot and calf, supply air to leg and/or foot chambers that are intermittently inflated with air to a 35 to 55 mm Hg pressure in a uniform or sequential fashion: 10 to 35 seconds [54]. This follows by 1 minute deflation period to allow the leg or foot to refill with blood [54]. These devices have been used prior to, during and following surgery to prevent DVT [53]. It has also been reported that combination of fondaparinux and intermitted pneumatic compression device was superior to pneumatic compression alone in reducing the rate of VTE in patients undergoing abdominal surgery [60].

#### 5.2.3. Inferior Vena Cava Filters

Inferior vena cava filters may be inserted through the infrarenal, jugular, femoral, or antecubital veins to relieve pulmonary vascular obstruction in patients with proximal DVT [53]. However, these filters do not prevent VTE in the lower extremities; hence, they should be combined with pharmacological interventions to reduce the risk of further development of thrombosis [53].

## 6. Concluding Remarks

In summary, VTE is a multifactorial disease with both environmental and genetic related risk factors. VTE is a significant threat to individuals that causes various compilations leading to death as well as financial burden for community. However, this condition can be diagnosed by various noninvasive cost-effective diagnostic algorithms in combination with noninvasive imaging techniques. Pharmacological and nonpharmacological interventions, either alone or in combination, can be used to prevent or manage this condition. In particular, new generation of anticoagulants to target a specific step in coagulation cascade and with the possibility to replace the common pharmacological treatments due to enhanced safety and reduced side effects are among the highlights of current investigations. Meanwhile, the role of nonpharmacological approaches that are easier to use with no risk of bleeding should not be neglected. The combination of both interventions may ease the process of prevention and treatment of VTE. However, more investigation is required to fulfil these goals.

## Figures and Tables

**Table 1:** Clinical characteristics for predicting the pretest probability of deep venous thrombosis. The Wells score model demonstrates well-established criteria for assessment of suspected DVT [22, 23].

Wells score	
Clinical characteristics	Score
Active cancer	+1
Paralysis or plaster immobilisation	+1
Bed rest >3 days or major surgery <4 weeks	+1
Localised tenderness along the distribution of the deep venous system	+1
Entire leg swollen	+1
Calf swelling >3 cm when compared with asymptomatic leg	+1
Pitting oedema	+1
Collateral superficial veins (nonvaricose)	+1
Previously documented deep vein thrombosis	+1
Alternative diagnosis at least as likely as deep vein thrombosis	−2

Clinical probability	

Unlikely	<2
Likely	≥2

**Table 2:** The main clinical scoring models for predicting the pre-test probability of pulmonary embolism. Well's score, revised Geneva and simplified revised Geneva are scoring systems for assessment of suspected PE [24–26].

Well's Score		Revised and simplified revised Geneva scores		
Clinical characteristics	Score	Clinical characteristics	Revised score	Simplified score
Haemoptysis	+1	Age >65 years	+1	+1
Cancer	+1	Active malignant condition	+2	+1
Previous pulmonary embolism or deep venous thrombosis	+1.5	Surgery or fracture within 1 month	+2	+1
		Haemoptysis	+2	+1
Heart rate >100/min	+1.5	Previous deep vein thrombosis or pulmonary embolism	+3	+1
Recent surgery or immobilisation	+1.5			
Clinical signs of deep venous thrombosis	+3	Unilateral lower-limb pain	+3	+1
		Heart rate 75–94/min	+3	+1
Alternative diagnosis less likely than that of pulmonary embolism	+3	Pain on lower-limp deep venous palpation and unilateral oedema	+4	+1
		Heart rate >94/min	+5	+1

Clinical probability		Clinical probability		

Low	<2	Low	0–3	0-1
Intermediate	2–6	Intermediate	4–10	2–4
High	>6	High	>10	≥5
